# Sapovirus in Water, Japan

**DOI:** 10.3201/eid1301.061047

**Published:** 2007-01

**Authors:** Grant S. Hansman, Daisuke Sano, You Ueki, Takahiro Imai, Tomoichiro Oka, Kazuhiko Katayama, Naokazu Takeda, Tatsuo Omura

**Affiliations:** * National Institute of Infectious Diseases, Tokyo, Japan; † Tohoku University, Sendai, Japan; ‡ Miyagi Prefectural Institute of Public Health and Environment, Sendai, Japan

**Keywords:** Sapovirus, RT-PCR, phylogenetic analysis, genotype, dispatch

## Abstract

Sapoviruses are etiologic agents of human gastroenteritis. We detected sapovirus in untreated wastewater, treated wastewater, and a river in Japan. A total of 7 of 69 water samples were positive by reverse transcription–PCR. Phylogenetic analysis of the viral capsid gene grouped these strains into 4 genetic clusters.

The family *Caliciviridae* contains 4 genera, *Sapovirus*, *Norovirus*, *Lagovirus*, and *Vesivirus*, which include sapovirus (SaV), norovirus (NoV), rabbit hemorrhagic disease virus, and feline calicivirus strains, respectively. SaV and NoV are agents of human gastroenteritis. The most widely used method of detection is reverse transcription–PCR (RT-PCR), which has a high sensitivity and can also be used for genetic analysis. Only a limited number of SaV studies have been conducted, although most studies have shown that SaV infections are more frequent in young children than in adults and that nearly all children are infected by 5 years of age.

NoVs have been detected in oysters (and other shellfish), water from drinking fountains, ice, and community drinking water (*1*–*4*). Environmental studies of SaV have not been conducted. SaV strains can be divided into 5 genogroups (GI–GV), among which GI, GII, GIV, and GV infect humans; GIII infects porcine species. Phylogenetic studies have also designated SaV clusters or genotypes to further describe strains that differ by ≈10% in nucleotide or amino acid sequences. The purpose of this study was to identify and describe SaV strains in environmental samples, namely, untreated wastewater, treated wastewater, a river, and seawater, in Japan.

## The Study

**Figure 1:**
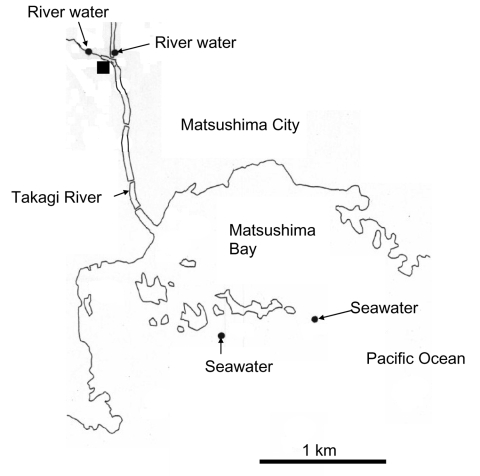
Locations in Miyagi Prefecture, Japan from which water was isolated. The solid square shows the location of the wastewater treatment plant (sampling site of untreated and treated wastewater).

Water samples were obtained at different locations once a month in Miyagi Prefecture, Japan, from March 14, 2004, through February 16, 2005 (*5*). A total of 69 samples were obtained, which included 12 untreated wastewater samples, 12 treated wastewater samples, 23 river samples (2 different locations), and 22 seawater samples (2 different locations) (Figure 1). Untreated wastewater and treated wastewater were obtained from a wastewater treatment plant that processes domestic wastewater from residents living in a nearby city (Matsushima City). The treated wastewater is chlorinated at the wastewater treatment plant and then discharged into the Takagi River. The river runs directly into Matsushima Bay and then into the Pacific Ocean. River water was obtained from 2 locations upstream from the wastewater treatment plant, and seawater was obtained from 2 locations outside Matsushima Bay in the Pacific Ocean.

The methods of viral concentration were different for each location, as previously described (*5*). For untreated wastewater, 1 L was centrifuged for 15 min at 9,000 × *g* and concentrated with polyethylene glycol (resuspended in 4 mL distilled water). For treated wastewater and river water, 1 L was directly concentrated with polyethylene glycol. For seawater, 10 L was filtered, viruses were absorbed to a filter (type HA negatively charged membrane with a 0.45-μm pore size, Nihon Millipore, Tokyo, Japan) and eluted in 40 mL alkali buffer, and 40 mL buffer was further concentrated by ultracentrifugation to give a final volume of 500 μL (*6*).

RNA was extracted as previously described (*7*). Nested RT-PCR was used to detect all human genogroups (*8*). For the first PCR, primers F13, F14, R13, and R14 were used. For the nested PCR, primers F22 and R2 were used. All RT-PCR products were analyzed by electrophoresis on 2% agarose gels and visualized by staining with ethidium bromide. RT-PCR products were excised from the gel and purified using the QIAquick gel extraction kit (QIAGEN, Hilden, Germany). Nucleotide sequences were determined with the terminator cycle sequence kit (version 3.1) and the ABI 3130 Avant sequencer (PerkinElmer Biosystems, Wellesley, MA, USA). Sequences were aligned with Clustal X (*9*), and distances were calculated by using the Kimura 2-parameter method as previously described (*10*). Nucleotide sequence data from this study have been deposited in GenBank under accession nos. DQ915088–DQ915094.

**Figure 2:**
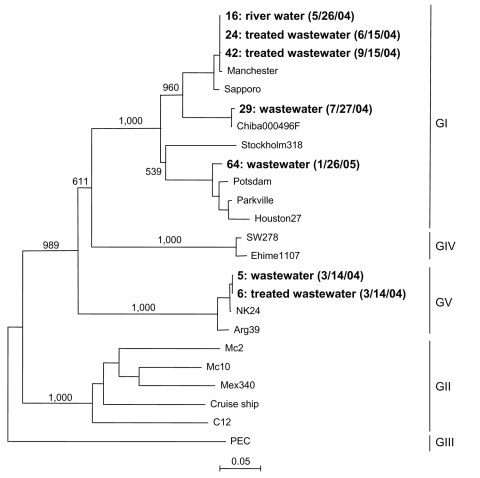
Phylogenetic analysis of sapovirus capsid nucleotide sequence showing different genogroups. Items in **boldface** are sequences isolated in this study and dates of isolation. Numbers on each branch indicate bootstrap values for the genotype. Bootstrap values >950 were considered statistically significant for the grouping. The scale bar represents nucleotide substitutions per site. Manchester, X86560; Sapporo, U65427; Chiba000496F, AJ412800; Stockholm318, AF194182; Potsdam, AF294739; Parkville, U73124; Houston27, U95644; SW278; DQ125333; Ehime1107, DQ058829; NK24, AY646856; Arg39, AY289803; Mc2, AY237419; Mc10, AY237420; Mex340, AF435812; cruise ship, AY289804; C12, AY603425; PEC, AF182760.

SaV was detected in 7 (10%) of 69 concentrated water samples. Negative controls were included in the RT-PCR and showed negative results (data not shown). Genetic analysis of the positive samples showed 4 distinct genetic clusters, which included 3 GI clusters and 1 GV cluster (Figure 2). Three GI sequences were identical (strains 16, 24, and 42), 2 of which were obtained from treated wastewater 3 months apart (strains 24 and 42), and 1 was obtained from the river water (strain 16). The other 2 GI sequences grouped into 2 different clusters (strains 29 and 64) and were isolated from untreated wastewater. The 2 GV sequences were identical (strains 5 and 6). These 2 GV-positive samples were obtained on the same day, although they were obtained from different locations, i.e., untreated wastewater and treated wastewater (Figure 1). Comparison of SaV sequences detected in this study with sequences in GenBank indicated that all 7 isolates closely matched previously reported SaV sequences (Figure 2). Positive SaV samples were obtained in both hot (summer) and cold (winter) months.

## Conclusions

Human SaVs infections are being detected more often worldwide (*7*,*11*,*12*). These novel results have shown that like NoV (*5*), SaV can also be detected in water samples. Most sequences detected in water samples (5 of 7) belonged to GI. This genogroup likely represents the dominant genogroup worldwide (*7*,*10*,*13*). Two sequences (strains 5 and 6) belonged to GV, which has not yet been reported in Japan.

In a similar study, NoV was detected from water samples from the same research locations (*5*). Detection of SaV in river water samples upstream from the wastewater treatment plant suggests human fecal contamination in the river and that SaVs persist in freshwater. Screening for SaV may be worthwhile in oyster samples because NoVs were detected in oysters from local oyster farms (*5*). However, the failure to detect SaV in seawater samples may indicate that the sampling sites were not affected by human fecal contamination or that SaVs do not survive in marine waters. Nevertheless, further environmental studies are clearly needed to address this issue.
